# Single-Atom Catalysis: How Structure Influences Catalytic Performance

**DOI:** 10.1007/s10562-019-02709-7

**Published:** 2019-02-25

**Authors:** Gareth S. Parkinson

**Affiliations:** Institute of Applied Physics, TU Wien, Vienna, Austria

**Keywords:** Single-atom catalysis, Surface science, Scanning tunneling microscopy, IRAS

## Abstract

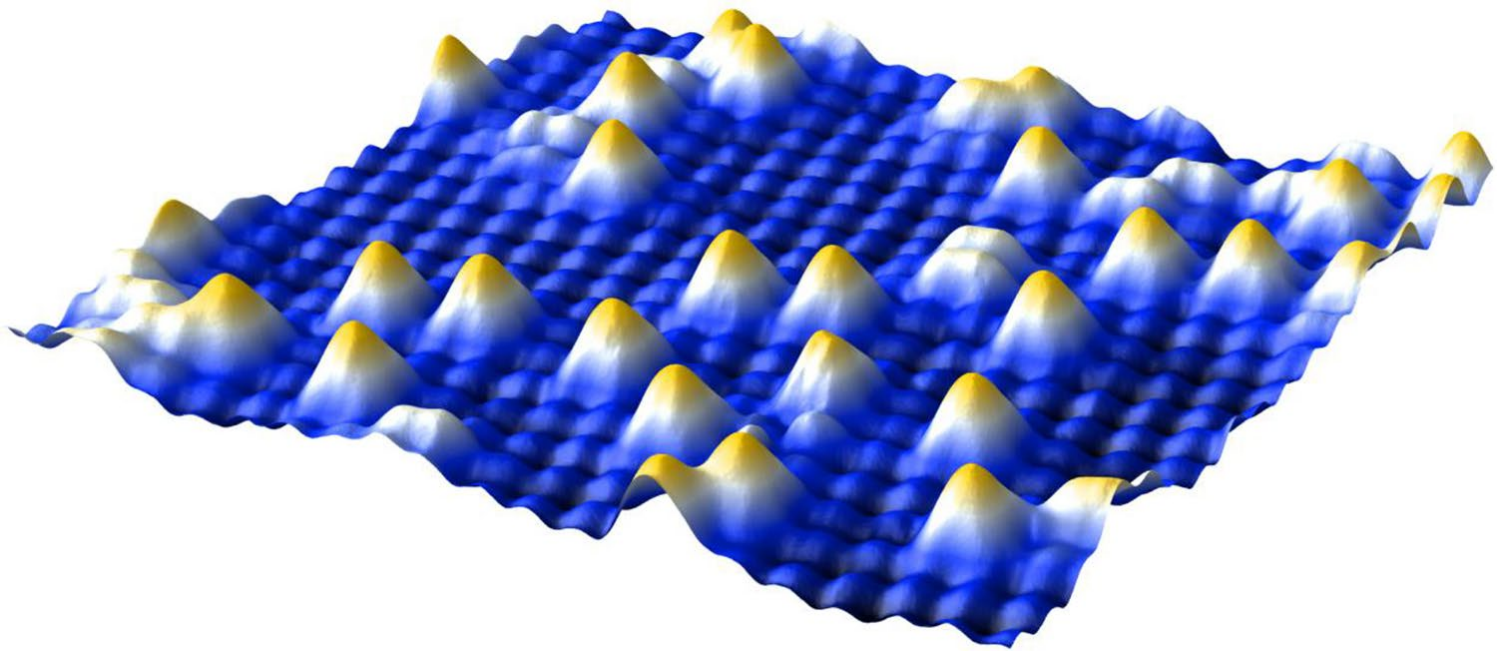

The field of “Single-atom catalysis” [1–7] came about as the ultimate limit of the ongoing effort to minimise the amount of precious metal required in heterogeneous catalysts. Although driven by economic considerations, the transition from nano and even subnano metal particles supported on inexpensive materials has yielded fascinating science, including the remarkable discovery that Au, the noblest of metals, becomes catalytically active in nano form [8]. Pioneering experiments with size-selected clusters have contributed significantly to our understanding of the size effect (see for example [9–12]), and such studies provided some of the earliest evidence that the smallest possible supported nanoparticle, an isolated adatom, could be catalytically active [13]. In 2003, Flytzani-Stephanopoulos and coworkers [14] went one step further: They noticed that an Au/ceria catalyst exhibited similar water-gas shift activity when the metallic nanoparticles were leached away, and concluded that strongly bound, ionic Au species were responsible all along. Over time, this phenomenon has been demonstrated to occur on other metal oxide supports including titania and iron oxide [15].

Today, many groups intentionally synthesize catalysts based solely on “single-atom” active sites. Not only does this make the most efficient use of precious metal, but if all active sites are the identical, selectivity problems associated with a inhomogeneous particle size distribution can be avoided. A common approach is to select a system for which nanoparticles are active, e.g. Pt for CO oxidation, and then create a stable “single-atom” variant. This is not straightforward, because a strong thermodynamic driving force causes adatoms to sinter into larger nanoparticles on metal oxide surfaces, but it is now routinely achieved. Comprehensive summaries of the different synthesis methods have been published recently [1, 16], and I will not attempt to summarize them here. I will, however, briefly describe the method of Zhang and coworkers, who coined the phrase “single-atom catalyst” to describe a Pt_1_/FeO_x_ system prepared by a wet chemical approach in 2011 [7]. Rather than leaching away the metallic component, their catalyst and support were co-precipitated from solution containing H_2_PtCl_6_·H_2_O and Fe(NO_3_)_3_·9H_2_O. Fine tuning of the temperature and pH was required to obtain the atomic dispersion, together with an extremely low Pt loading [0.17 weight percent (wt%)]. Samples with a slightly higher loading (2.5 wt%.) already exhibited Pt nanoparticles [7]. Following extraction, drying, and calcination (primarily to remove the precursor ligands), the “single-atom” catalyst exhibited excellent low-temperature CO oxidation activity compared to both Pt nanoparticles and a nano-Au standard. Based on density functional theory (DFT) calculations, the authors proposed the unique catalytic activity originated in the relatively weak binding of CO to cationic Pt adatoms. A Mars–van Krevelen (MvK) mechanism was proposed, whereby the oxygen to oxidise CO is initially extracted from the support lattice, and the resulting oxygen vacancy subsequently repaired by O_2_ supplied from the gas phase [7]. Note that this differs significantly from CO oxidation on Pt nanoparticles, where molecular O_2_ dissociates on neighbouring Pt sites facilitating a direct reaction with CO. Another novel approach, pioneered by Datye and Wang, is to trap highly mobile Pt adatoms undergoing Oswald ripening at high binding energy sites on ceria [5, 17]. The resulting catalysts are remarkably stable and effective for CO oxidation, and interest in the field continues to grow at a rapid pace (Fig. 1).

**Fig. 1:**
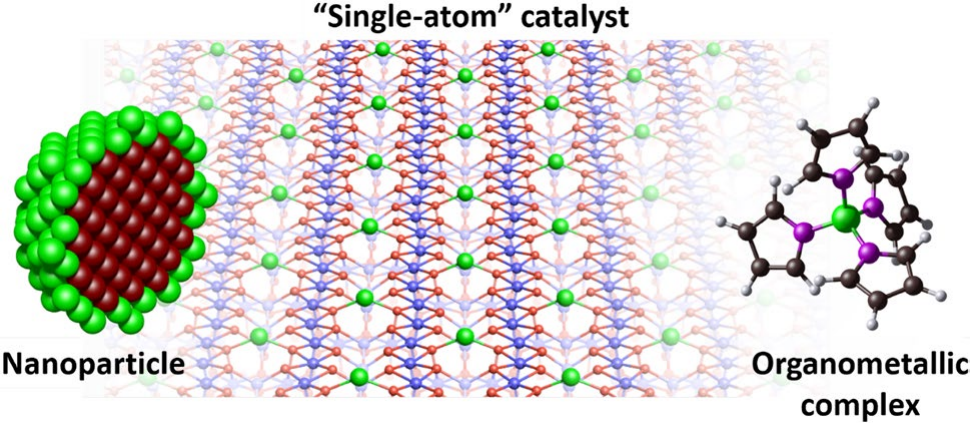
Single-atom catalysis represents the ultimate limit to the downsizing of precious metal nanoparticles. In the ideal scenario, all metal atoms can be utilized for reactions, and all active sites are identical. The charged metal centre bound to surface atoms has seen SAC systems likened to the organometallic complexes utilized in homogeneous catalysis. Figure adapted from Ref. [18]

It is important to note, however, that the concept of single-atom catalysis has been controversial. Ding et al. [19], for example, published a paper in which they claimed that isolated Pt atoms are actually inactive for both low-temperature CO oxidation and water–gas shift chemistry, and that any catalytic activity probably arose from metallic Pt nanoparticles. Infrared reflection absorption spectroscopy (IRAS) data were presented to show that CO binds more strongly to cationic Pt than metallic Pt clusters, not weaker as Qiao et al. [7] had proposed. In both these works, the as-prepared catalysts were imaged using scanning transmission electron microscopy (STEM), as has become the standard in experimental SAC studies [20]. The resolution of this technique has progressed tremendously over recent years, and adatoms and clusters can be distinguished from the support by virtue of a strong atomic number contrast in high-angle annular dark-field (HDAAF) mode [20]. In both cases, IRAS data exhibited a CO stretch above 2100 cm^−1^, consistent with binding to cationic Pt [21], while X-ray absorption near edge structure (XANES) data suggested that Pt was most likely coordinated to oxygen, with no sign of Pt–Pt bonding. The directly opposing conclusions of these two studies highlight a major issue in the SAC field today; it is extremely difficult to unambiguously prove the active species in catalysts based on adatoms and subnano clusters. Even if a catalyst is shown to be atomically dispersed initially, it may evolve in operando and there is no guarantee it remains in this state when reactivity is measured.

Recent work from Phil Christopher’s group appears to shed new light on the origin of the controversy discussed above. In this work [22, 23], extremely low Pt loadings (0.05 wt%) were deposited onto 5 nm anatase TiO_2_ nanoparticles in solution such that, on average, each particle contained just one Pt adatom. Thus, the risk of in situ sintering is substantially reduced. Following calcination in flowing air at 450 °C for 4 h to remove ligands from the Pt-precursor, an extremely sharp absorption band was observed at 2112 cm^−1^ in IRAS, and assigned to cationic Pt occupying a highly homogeneous adsorption site (see Fig. 2). Interestingly, however, temperature programmed desorption (TPD) experiments showed that the CO desorbs from such samples already at 5 °C, in apparent agreement with the conclusion of Qiao et al. [7]. Samples prepared with a higher loading exhibited the behaviour expected for Pt clusters, a broad band below 2100 cm^−1^ and CO desorption well above room temperature. Crucially, when the clustered samples were pre-oxidised prior to CO adsorption, the CO stretch shifted back above 2100 cm^−1^, indicative of cationic Pt, but the CO remained adsorbed up to 300 °C in TPD. The authors therefore conclude that weakly bound CO is associated with cationic adatoms, and strongly bound CO with cation-like CO stretch is due to adsorption on small PtO_x_ clusters. This discussion highlights that the local binding environment plays a defining role in the strength of the interaction with CO, not the charge state. It is thus insufficient to attribute catalytic activity to Pt^2+^ species, for example, because many different geometries can coexist with similar charge state, and perhaps only one might be catalytically active. In any case, assigning charge states of Pt based on the CO stretch is problematic, as the measured shift has been shown to depend on the local geometry [21]. To make the most of SAC, we need to determine the atomic-scale structure of the optimal active site, and then figure out how to promote this on the catalyst.

**Fig. 2:**
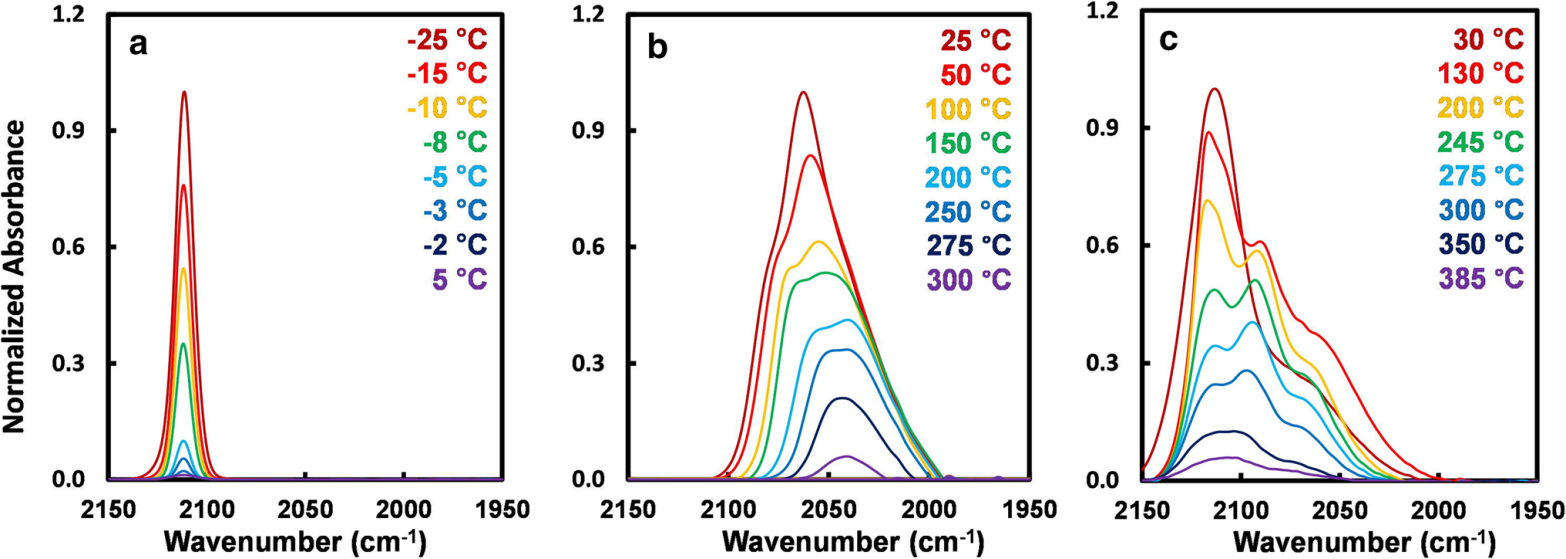
CO TPD-IR spectra from catalysts based on Pt adatoms (**a**), Pt clusters (**b**) and oxidised Pt clusters (**c**) supported on nano-TiO_2_. The CO stretch from Pt_1_ adatoms at 2113 cm^−1^ is extremely sharp, suggestive of a homogeneous adsorption site, and CO is weakly bound. CO adsorbed on oxidised Pt clusters exhibits a similar peak position, but the band is significantly broader. CO binds most strongly to oxidised Pt clusters. Figure reproduced with permission from Ref. [23]

Unfortunately, precise determination of the active site geometry on a real single atom catalyst is extremely difficult. From STEM experiments, we do know that the heavy metal atoms appear congruent with the cation lattice of the metal-oxide support, which suggests coordination to oxygen. This is consistent with XANES data, and DFT calculations, which generally find greater stability with coordination to oxygen. Unfortunately, the lighter oxygen atoms are not well imaged in STEM, and even if they were, any surface specific rearrangement of the lattice around the adatom would be impossible to discern because the images are a 2D representation of the 3D object. For a full discussion of the strengths and weaknesses of STEM for SAC studies, see ref [20]. Although some information on the coordination number can be derived from XANES, reliable interpretation requires significant homogeneity on the sample, which is probably not the case on a real catalyst, and relevant reference data for comparison. In general, it is probably fair to say that the primary source of local structural information in SAC is DFT-based calculations.

The theoretical approach to modelling SAC typically takes low-index surfaces as representative of the support. In their extensive studies of “FeO_x_” supported SACs, for example, Li and coworkers [24] utilize an oxygen terminated α-Fe_2_O_3_(0001) surface, and adsorb metal adatoms where the next Fe cation would reside if the bulk structure were continued. This adsorption site is in qualitative agreement with the STEM results described earlier [20], but it is important to note that there is no direct evidence from experiment that the support has this particular stoichiometry or structure following calcination. Since the real support material consists of small nanoparticles it likely exhibits a high density of different step, kink, and point defects that might be expected to bind adatoms more strongly than regular terrace sites. Moreover, the presence of adsorbates such as water and hydroxyls is not considered, nor the possibility of any remnant ligands from the liquid phase preparation. Nevertheless, such calculations demonstrate that it is plausible for CO oxidation to occur via a Mars–van Krevelen type mechanism at the temperatures observed experimentally, with the rate limiting step linked to extraction of lattice oxygen from the support to form CO_2_. Recently, Li’s group performed a systematic study of M_1_/FeO_x_ SACs (M = Pt, Pd, Ni, Fe, Cu, Co, Ru, Rh, Ag, Os, Ir, Au) for CO oxidation [24], and concluded that a high CO adsorption energy correlates with a high barrier for O_lattice_ extraction. This is clearly something that could be tested in TPD experiments with a well-chosen model system, as will be discussed in what follows. Interestingly, Pd_1_ and Ni_1_ are predicted to perform at least as well as Pt_1_ for CO oxidation, with rate determining steps significantly lower than 1 eV. Since the properties of adatoms do not scale from those of nanoparticles, it is not clear that Pt need be the best adatom metal for CO oxidation. Thus, instead of merely reducing the amount of Pt required, using isolated adatom geometries may provide a route to eliminate it entirely in some cases.

In a similar computational screening study, Li et al. [25] independently investigated the FeO_x_ supported system and proposed that Rh_1_, Pd_1_, Ru_1_ and even Ti_1_ and Co_1_ have the potential to outperform Pt_1_ for CO oxidation via a Langmuir Hinshelwood-type mechanism. In this process, CO and O_2_ initially adsorb on the same adatom (see step E2, Fig. 3), before (step E5) CO_2_ desorbs having reacted with one O atom from the adsorbed O_2_ molecule. In step E7, a second CO adsorbs to react with the remnant O atom. Such a mechanism has yet to be confirmed experimentally, but a stable Rh_1_(CO)_2_ dicarbonyl species has been observed in the Rh_1_/α-Al_2_O_3_ SAC system [26]. The ability to adsorb multiple molecules simultaneously to the same adatom will likely depend strongly on its coordination to the support, and is key to the idea that SAC systems can function like homogeneous catalysts.

**Fig. 3:**
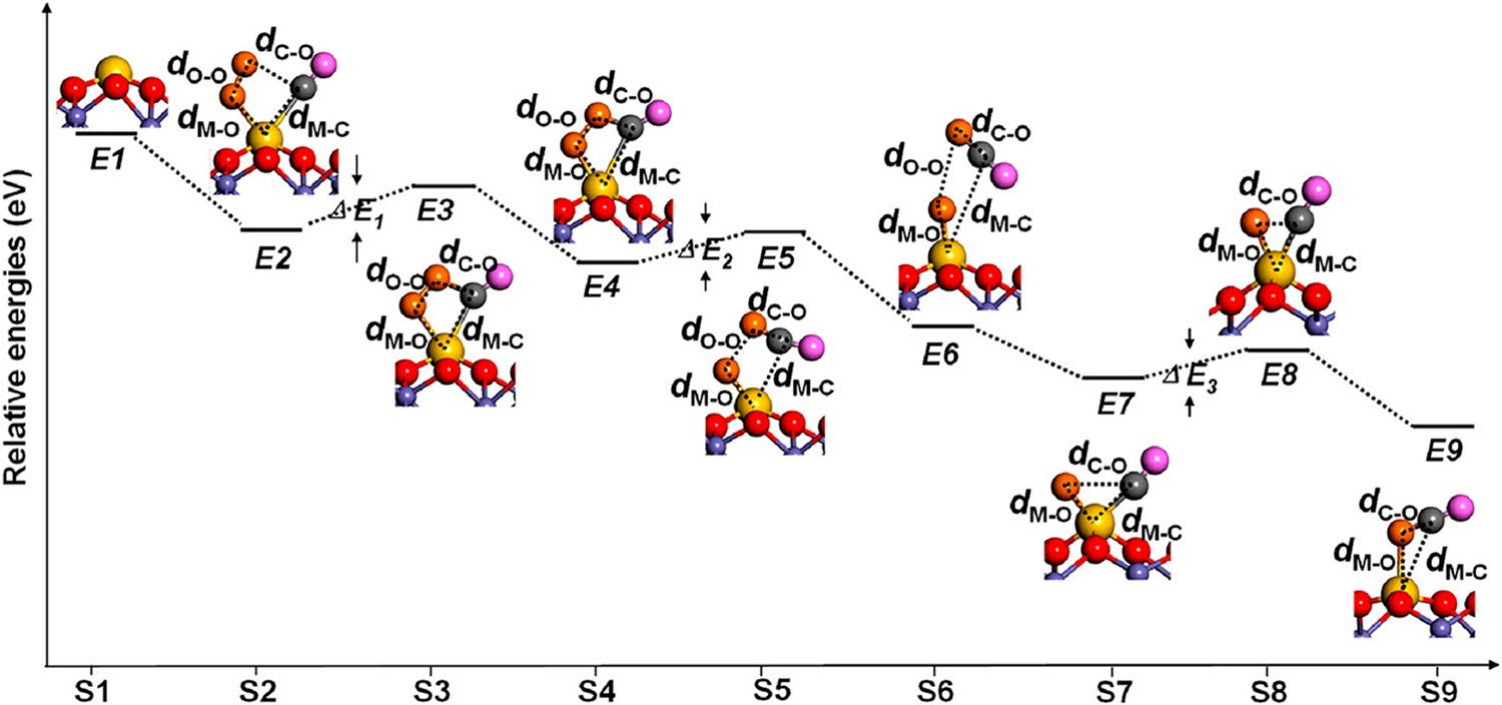
Theoretically predicted reaction pathway of CO oxidation on M_1_/Fe_2_O_3_(0001) (M = Ru, Rh, Pd, Co, and Ti) via the Langmuir–Hinshelwood mechanism. Color scheme: M, gold; C, grey; O of O_2_, orange; O of CO, pink; O of the iron-oxide support, red; Fe, purple. Figure reproduced with permission from Ref. [25]

To better understand the role of coordination in SAC it makes sense to perform experiments on model single-atom catalysts, where the structure of the support and the geometry of the adatom can be precisely determined. The so-called “surface science” approach, based on single-crystal supports and tightly controlled conditions, has contributed significantly to our understanding of heterogeneous catalysis, but as yet there have been relatively few studies addressed to SAC. One reason for this is that metal adatoms are known to rapidly agglomerate when metal is evaporated onto a model metal-oxide support—usually a clean, low index single-crystalline surface—under ultrahigh vacuum (UHV) conditions. This already suggests that the stable adatoms encountered in SAC are most likely not the adatoms adsorbed at regular lattice sites often assumed in theoretical calculations. Indeed, Datye and coworkers [5, 17] specifically utilize such high binding energy sites in their recent work to trap mobile Pt species emitted from larger nanoparticles on ceria at elevated temperature, and the resulting catalyst is highly active for CO oxidation. There is substantial evidence from surface-science experiments and theoretical calculations that Pt^2+^ adatoms are extremely stable in highly coordinated at PtO_4_ centres at steps on CeO_2_(111) [27]. Defects such as oxygen vacancies, cation vacancies, and steps are clearly very important, and the ability to identify and even selectively create different defects by surface scanning probe microscopies makes this approach ideal to study the phenomenon using surface-science techniques in the future.

In this author’s group, the Fe_3_O_4_(001) surface is utilized as a model support. A unique subsurface reconstruction essentially creates an array of strongly binding defect sites (periodicity 0.84 nm), on which metal adatoms of almost any variety bind with a twofold coordination to lattice oxygen [28]. The DFT-determined structure has been confirmed by quantitative low-energy electron diffraction (LEED) [28] and surface X-ray diffraction (SXRD) [29] experiments, and the adatom geometry inferred from scanning tunnelling microscopy images was verified by normal incidence X-ray standing waves (NIXSW) experiments and DFT calculations [30, 31]. A major benefit of this model system is that the stability and reactivity of different metals can be compared in a direct manner. So far, we have found that 3d transition metals are extremely stable against thermal sintering on Fe_3_O_4_, but tend to incorporate into the surface layer at elevated temperature [32]. This is easy to understand because these metals form stable MFe_2_O_4_ ferrite compounds, and thus transition from the twofold coordination to a more stable octahedral site when sufficient thermal energy is supplied [32]. Metals such as Au [33], Ag [34] and Pt [35] do not incorporate in the spinel lattice, and remain stable as adatoms until the reconstruction is lifted thermally at 700 K [36]. Our DFT calculations suggest that the stability of the adatom phase is not due to a lack of mobility, but rather because the dimers tend to be highly unstable on this surface, which prevents formation of a critical cluster nucleus [34, 35]. Thermal stability is drastically reduced in the presence of pre-existing nuclei, however, which efficiently capture mobile adatoms and rapidly grow in size.

Recent HDAAF-STEM experiments on real SAC systems have observed enhanced Pt_1_ mobility in reactive gas atmospheres [37]. One of major conclusions of our work on Fe_3_O_4_(001) is that CO adsorption destabilizes otherwise stable adatoms by weakening their interaction with the substrate. Figure 4a shows a Pt_1_ adatom as calculated by DFT + U, and panels B and C show the effect of CO adsorption. The double lobed appearance of Pt_1_CO in STM in paned C is due to two equivalent geometries with a small barrier for switching. Figure 4c shows selected frames from an STM movie, in which the CO-induced sintering of 4 adatoms into a Pt_4_ cluster was directly observed. STM, and increasingly noncontact AFM [38, 39], are extremely powerful tools and ideal for tracking surface specific processes with exquisite detail. Since this technique relies on having a relatively flat support, applying it directly to nano-sized supports ‘real’ SACs is not an option.

**Fig. 4:**
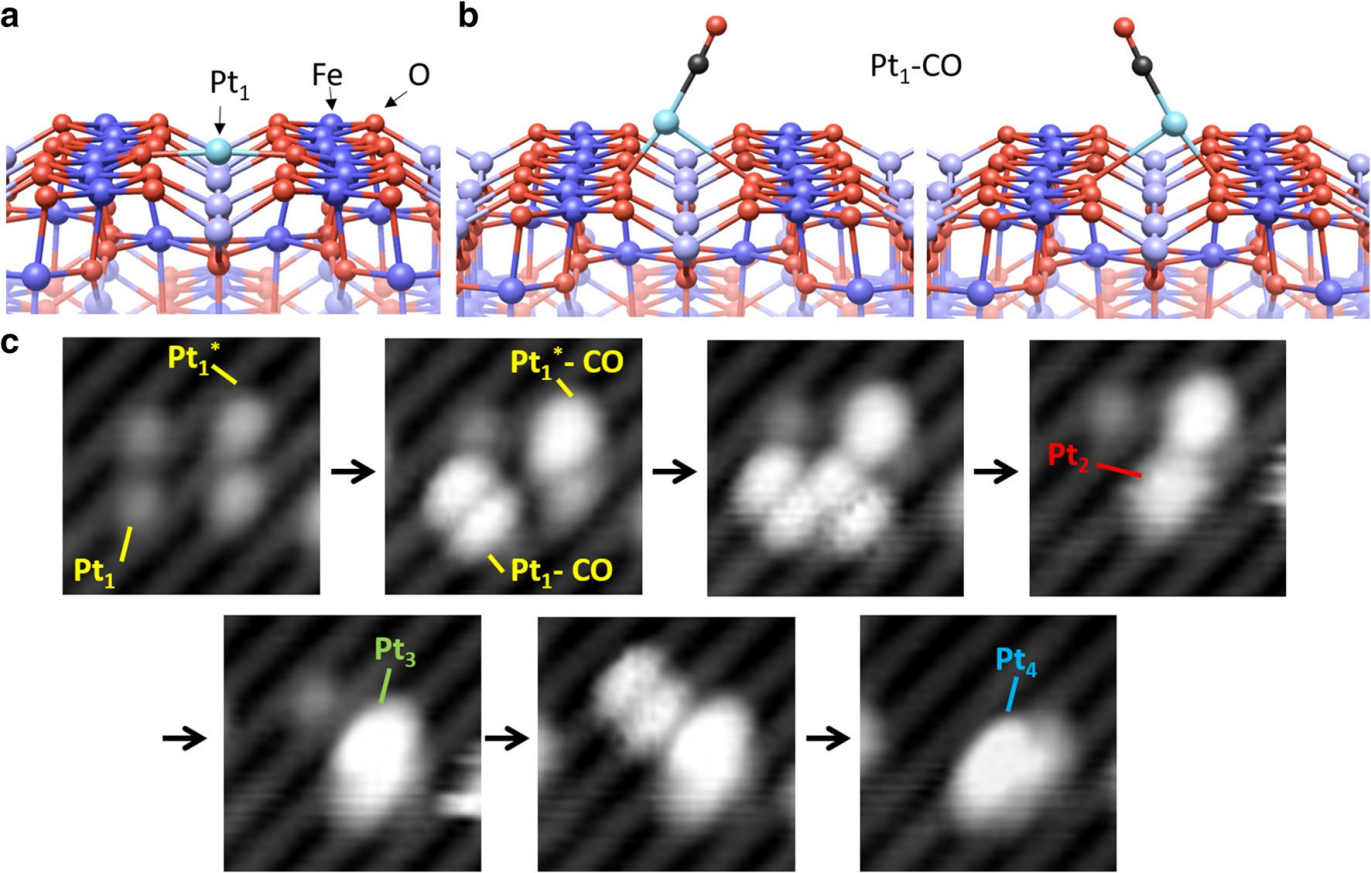
CO-induced Pt diffusion and coalescence on Fe_3_O_4_(001)−(√2 × √2)R45°. **a** The energetically preferred Pt_1_ geometry is twofold coordinated to surface oxygen atoms across neighboring rows (A metastable geometry (Pt1*) in which Pt is bound to two O atoms along the row is not shown). **b** Adsorption of a CO molecule lifts the Pt atom from the surface, resulting in a Pt–CO species that switches rapidly between two symmetrically equivalent configurations at room temperature. This produces a double-lobed appearance in STM. **c** STM image sequence acquired during exposure to 2 × 10^−10^ mbar CO showing the formation of a Pt tetramer: CO adsorption on Pt_1_ and Pt_1_*, mobility, and coalescence are observed atom by atom. Figure adapted from Ref. [40] with permission from the authors

In the case of Pd/Fe_3_O_4_(001) [41], CO adsorption leads to rapid sintering into large particles, but for Pt, a large proportion (≈ 50%) of stable Pt_2_ dimers are obtained (an example is labelled Pt_2_ in Fig. 4c). The Pt_2_ dimers remain as long as the CO is adsorbed, and decay back into two adatoms when the system is heated above 500 K [35]. Interestingly, we find from XPS and DFT that the Pt_1_ adatoms are close to neutral on the surface, but CO adsorption leads to significant electron transfer into the substrate. Thus, an IRAS experiment would have suggested a cationic charge state. This observer effect has been reported previously for Au adatoms on MgO [42], and it is thus important to recognise that CO adsorption can significantly modify the electronic properties of a system, as well as induce reconfiguration or diffusion.

To study the effect of coordination, it would be interesting to compare the properties of metal adatoms on other facets of Fe_3_O_4_. For example, a prior STM investigation has shown that Au adatoms are likely cationic and stable at room temperature under UHV conditions on Fe_3_O_4_(111), and CO adsorption was observed at 260 K [43, 44]. Theoretical calculations predict strong binding of various metals in a threefold coordinated site on this surface [45], and this site is similar to the geometry utilized by Li et al. [25] for their calculations of FeO_x_. It would be fascinating if their predictions regarding coadsorption could be verified on a model system, and if so, it would be desirable to collect benchmark IRAS spectra to allow such species to be detected in real SAC systems. One final note on iron oxides; the Fe_3_O_4_(111) surface mentioned above was prepared by mild reduction of a α-Fe_2_O_3_(0001) single crystal [43, 44], which demonstrates the ease with which the iron oxides transition between different stoichiometry at the surface in different environments. In general, it would be interesting to learn how calcination affects the structure and stoichiometry of UHV-prepared surfaces, and whether the stability and reactivity of metal adatoms is modified.

The Sykes group at Tufts University have focussed on an altogether different model system, employing a Cu_2_O(111)-like thin film grown on Cu(111) as a model support. Their combination of STM, DFT and IRAS data clearly show that isolated adatoms predominate at low coverage in this system, and isotopically labelled TPD spectra conclusively demonstrate that CO oxidation occurs at 345 K via a MvK mechanism. Following the TPD, the system deactivates because Pt diffuses under the oxide skin and alloys with the Cu. Interestingly, the CO stretch associated with single atoms appears below 2100 cm^−1^, and this, combined with XPS and DFT evidence, led the authors to conclude the Pt adatoms are adsorbed in a neutral charge state. As discussed above, it is generally thought that Pt adatoms derive their stability from bonds to oxygen, and thus become cationic. Studies on model systems, however, show this is not always the case. It must be noted that the Pt adatom geometry on the Cu_2_O thin film, proposed on the basis of STM and DFT data, has Cu nearest neighbours and the metallic Cu bulk in close proximity, and thus is perhaps not representative of the bulk metal oxides typically used as catalyst support. It does show however, that there are interesting alternatives to be explored. Since Mars–van Krevelen type reactions seem prevalent in such systems, it makes sense to investigate highly reducible supports, such as surface oxides, where oxygen is weakly bound.

In this authors opinion, one aspect of SAC that requires detailed study is the role of water. In a follow up the their work on Pt/TiO_2_ [23], Christopher’s group collaborated with Pacchioni and coworkers to investigate the structural motif that might be responsible for the isolated Pt adatoms causing the extremely sharp CO stretch observed in Fig. 2a. Based on a comparison of theoretically calculated vibrational frequencies, they conclude that geometries based on the stable anatase TiO_2_(101) termination, with or without steps, cannot explain the observed vibrational and thermal stability of the adsorbed CO. Incorporation within the lattice, which might be expected to underlie the high stability of the isolated Pt species, is also incompatible with the experimental data. The best agreement was achieved when additional O atoms are included in the geometry, which the authors propose can be supplied from the large amount of surface OH groups that will be omnipresent on the surface under operating conditions.

Another hint of the importance of water comes from the recent work of Datye and coworkers [46], who have shown that pre-treatment of a Pt/CeO_2_ catalyst with steam results in a highly active SAC system. They propose OH bound at Pt^2+^ centres to be directly involved in the CO oxidation mechanism, and their DFT-determined reaction pathway (shown in Fig. 5) has a rate limiting energy barrier of 52 kJ mol^−1^. Initially, one O_lattice_H species coordinated with a Pt atom (Pt^2+^) reacts with CO adsorbed on Pt, creating an oxygen vacancy. This is then filled by adsorption of an oxygen molecule, and CO_2_ is generated via the deprotonation of the carboxyl intermediate; a process assisted by the newly adsorbed oxygen molecule. Subsequently, the OOH species (intermediate V) reacts with the second adsorbed CO, generating another CO_2_ molecule with a smaller activation barrier (TS3). Finally, the atomically dispersed surface is recovered after CO_2_ desorption. Such a mechanism is in line with the experiments of Wang et al. [47], who used isotopic labelling experiments to show that a significant proportion of the CO_2_ created by a Pt_1_/CeO_2_ catalyst contains oxygen supplied from water, and not from the oxide. They also propose CO to react with a hydroxyl to form a carboxyl intermediate, which then dehydrogenates with the help of a lattice hydroxyl to generate CO_2_. Sykes, McEwen and coworkers recently showed that water readily dissociates at Pt cations in their Pt/Cu_2_O model system [48].

**Fig. 5:**
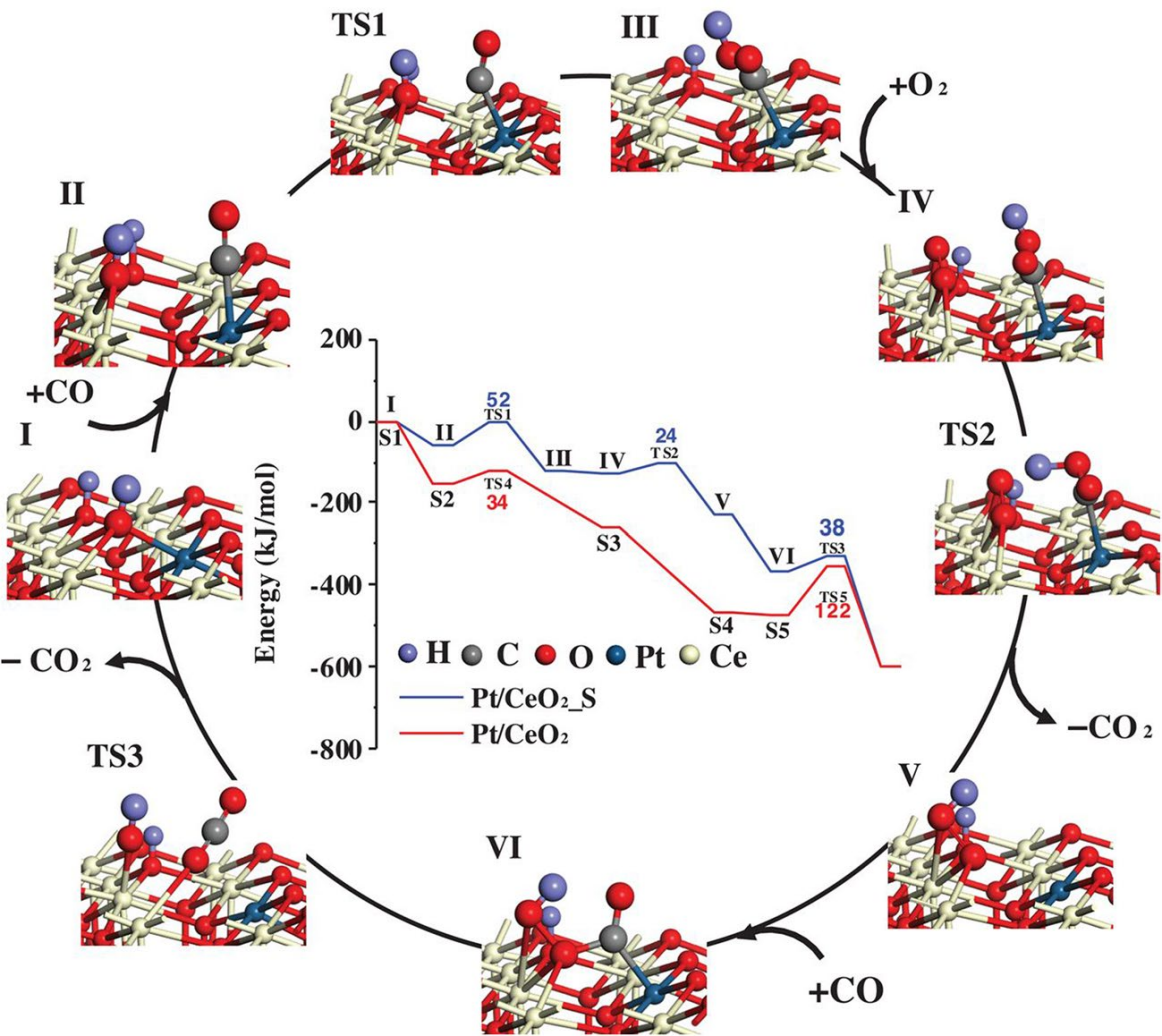
Proposed reaction mechanism for CO oxidation on the steam treated Pt/CeO_2_(111) surface. The structures of intermediates and transition states (TSs) correspond to the blue energy profile shown by the blue line in the inset. A legend for the atom colours is shown in the inset. Figure reproduced with permission from Ref. [46]

Clearly, the presence of water in the environment must be considered in models of “real” SACs, and understanding the effect of water on the CO oxidation reaction mechanism is a realistic target for model studies. In the present authors view, the anatase TIO_2_ model system is particularly attractive because preparation of a clean well-ordered anatase TiO_2_(101) surface is relatively straightforward under UHV conditions [49], oxygen vacancies can be created at will [50], the adsorption of water is well characterized [51, 52], and it is already clear that Pt adatoms are not stable at regular lattice sites [53]. With all these ingredients at hand, it should be possible to validate whether the controlled introduction of water has a direct impact on the adsorption and thermal stability of Pt adatoms, and to study if and how CO oxidation proceeds.

While CO oxidation is considered an ideal probe reaction to study the fundamentals of catalysis [54], it is certainly not the only reaction where SAC could make a significant impact. Liu and Corma have published an insightful summary of the potential applications in a variety of reactions [55], and highlight water–gas shift, selective hydrogenations, photocatalysis and electrocatalytic reactions as attractive targets. Isolated atom geometries are known to be useful for hydrogenation reactions in closely related, so-called single atom alloys (SAA) [56], where incorporated Pt and Pd atoms, for example, dissociate H_2_ leading to spillover onto a more inert host metal where further chemistry takes place [57, 58]. Zhang and coworkers have shown that this concept extends to metal oxide supports, and specifically that Pt_1_/FeO_x_ and Ir_1_/FeO_x_ can catalyse the selective hydrogenation of nitroarenes [59]. Clearly then, it is important to get a grasp of which metals best perform in the relevant processes, which is H_2_ activation in this case. The authors also note that Pd_1_/FeO_x_ and Rh_1_/FeO_x_ are as reactive as Pt and Ir, but suffered from a lack of selectivity. This suggests that the adatom also interacts with the nitroarenes, and the authors claim the excellent performance of Pt_1_/FeO_x_ is related to preferential adsorption of nitro groups. Similarly excellent performance was reported recently for Pd_1_ adatoms anchored on graphitic carbon nitride [60] and other carbon supported systems [61]. The only surface science work on this topic so far comes from Libuda and coworkers [62], who determined that the fourfold coordinated Pt^2+^ at step edges on CeO_2_(111) are inactive for H_2_ dissociation. Again, it is reasonable to expect that charge state and coordination of an adatom would play a role in this process.

The importance of the electrocatalytic reactions for emerging energy-related applications cannot be overstated. Lowering the required overpotential remains the bottleneck for a number of important technologies including water splitting and artificial photosynthesis. There has been interest in testing “single atom” electrocatalysts, and while there is evidence these systems can perform well, the mechanism of this reaction is not well understood and the reason for the catalytic activity is not clear. At present, the field electrochemical surface science on oxides remains in its infancy, but there are signs that it is becoming possible to perform electrochemical measurements on UHV prepared samples with a good degree of control [63]. As this line of research continues to mature, testing the stability and activity of the SAC model systems discussed above presents an opportunity to make a significant impact in an important and rapidly expanding area.

Over the last few years, researchers have noted a similarity between SAC and homogeneous catalysts [64, 65], because the charge state of the metal and its coordination sphere seem to define catalytic activity. There is much excitement at the prospect that SAC could bridge the gap between these fields [64], with isolated adatom active sites providing the extreme selectivity typical of homogeneous catalysts. The idea is then to target reactions typically performed by organometallic compounds in solution, and try to “heterogenize” the reaction using the same metal. Both Lang et al. [66] and Wang et al. [67] have demonstrated oxide supported Rh-SACs can catalyse hydroformylation of olefins as well as Rh-based homogeneous catalysts. In the latter case, the authors proposed an intriguing reaction pathway in which H_2_ dissociation occurs at Rh adatoms leading to surface OH groups, which take part in the reaction with CO and propene coadsorbed at Rh_1_. While most systems still prefer oxide supports, an alternative approach is to try to tailor supports to mimic the active centre of homogeneous catalysts. Chen et al. [68] anchored Pd on a graphitic carbon nitride support to mimic the coordination environment in Pd acetate, which is usually used to catalyse Suzuki couplings in solution. The result was a highly effective catalyst, which the authors attribute to the ligands, which provide an adaptive coordination of the palladium, and participates in the adsorption, stabilization and activation of the intermediates.

Clearly, the ability to tailor the atomic-scale structure of the active site is going to be key going forwards in the field of SAC. Ideally then, the extensive literature in homogeneous catalysis should serve as inspiration for which systems to study, but O^2−^ is not a particularly common ligand in organometallic complexes [69]. If we wish to keep using metal oxide supports, it is vital to learn how O^2−^ coordination affects adsorption processes and reactivity. Moving away from oxides, there are many examples of single atom catalysts supported by carbon, carbon nitride and other materials [2], but as yet there is relatively little surface science work investigating the catalytic properties of metal adatoms adsorbed on these supports. One exciting avenue is combining SAC with 2D materials, in particular graphene, g-C_3_N_4_, and MoS_2_, with the aim to modulate the reactivity of metal atoms through unusual bonding environments and electronic environments. In the current author’s opinion, SAC presents an exciting opportunity for surface science experiments to work hand in hand with theory to separate fact from fiction, and help determine what possibilities exist in this exciting area of research.
